# Advertising of unhealthy foods and beverages around primary and junior high schools in Ghana's most urbanized and populous region

**DOI:** 10.3389/fpubh.2022.917456

**Published:** 2022-09-23

**Authors:** Gideon Senyo Amevinya, Stefanie Vandevijvere, Bridget Kelly, Seth Kwaku Afagbedzi, Richmond Aryeetey, Akosua Pokua Adjei, Wilhemina Quarpong, Akua Tandoh, Silver Nanema, Charles Agyemang, Francis Zotor, Matilda E. Laar, Kobby Mensah, Dennis Laryea, Gershim Asiki, Michelle Holdsworth, Amos Laar

**Affiliations:** ^1^Department of Population, Family and Reproductive Health, School of Public Health, University of Ghana, Accra, Ghana; ^2^Sciensano, Service of Lifestyle and Chronic Diseases, Brussels, Belgium; ^3^Early Start, School of Health and Society, University of Wollongong, Wollongong, NSW, Australia; ^4^Department of Biostatistics, School of Public Health, University of Ghana, Accra, Ghana; ^5^Nutrition and Health Sciences, Laney Graduate School, Emory University, Atlanta, GA, United States; ^6^Department of Public and Occupational Health, Amsterdam University Medical Center (UMC), University of Amsterdam, Amsterdam, Netherlands; ^7^Department of Family and Community Health, University of Health and Allied Sciences, Ho, Ghana; ^8^Department Family and Consumer Sciences, School of Agriculture, University of Ghana, Accra, Ghana; ^9^Department of Marketing and Entrepreneurship, University of Ghana Business School, University of Ghana, Accra, Ghana; ^10^Non-communicable Disease Programme, Ghana Health Service, Accra, Ghana; ^11^African Population and Health Research Center, Nairobi, Kenya; ^12^UMR MoISA (Montpellier Interdisciplinary Centre on Sustainable Agri-Food Systems), (Univ Montpellier, CIRAD, CIHEAM-IAMM, INRAE, Institut Agro, IRD), Montpellier, France

**Keywords:** food environments, food advertising, unhealthy foods, schools, children, Ghana

## Abstract

**Introduction:**

The advertising of energy-dense, nutrient-poor foods and beverages is a common feature in obesogenic food environments. Such advertising, within and around settings where children live, learn, and play, negatively affects their food acquisition and consumption. We examined the extent and nature of food and beverage advertising around primary and junior high schools in Ghana's most populous and urbanized region, Greater Accra.

**Materials and methods:**

Outdoor advertisements for foods and beverages within a 250 m road network distance of 200 randomly sampled schools were geocoded. For each food and beverage advertisement, information was collected on the setting, type, size, and number of product types featured in the advertisement. Promotional techniques (promotional characters and premium offers) used in advertisements were documented. Advertised foods and beverages were classified using the INFORMAS and NOVA food classification systems.

**Results:**

A total of 5,887 advertisements were identified around the schools surveyed, 42% of which were for foods and beverages. Advertisements were most prevalent at food outlets (78% of all food advertisements), but also along roads and on non-food structures. Overall, 70% of food advertisements featured non-core/unhealthy products, while 12 and 14% had core/healthy and miscellaneous (including soup cubes, seasonings, and tea) products. About 4% of food advertisements had only a product/brand name or logo displayed. One out of two of the foods and beverages advertised were ultra-processed foods, 30% processed, 3% processed culinary ingredients, and 17% unprocessed or minimally processed foods. Sugar-sweetened beverages were the most advertised food product type (32%). Promotional characters were found on 14% of all food advertisements (most–69% were cartoons or manufacturer's characters), while 8% of all food advertisements had premium offers (including price discounts and gift/collectables).

**Conclusions:**

There is an abundance of unhealthy food advertisements around primary and junior high schools in the Greater Accra Region. Policy actions such as restricting the promotion of unhealthy foods in children's settings are needed to protect pupils from such advertising practices.

## Introduction

Preventing overweight and obesity among children is a global public health priority. Since the 1980s, childhood overweight and obesity have risen worldwide (1, 2). An increase from 4% in 1975 to over 18% in 2016 was reported among school children aged between 5 and 19 years globally (3). Diverse studies have identified exposure to unhealthy food environments as a major determinant of overweight and obesity, especially among countries undergoing what is termed the nutrition transition (4–6). Ghana is at an advanced stage of the nutrition transition; there is a rapid shift toward consumption of energy, fat, sugary, and salty foods and low levels of physical activity (7, 8).

A known factor driving the preference, acquisition, and consumption of unhealthy foods is food and beverage marketing (9, 10). The World Health Organization (WHO) defines marketing as “any form of commercial communication or message that is designed to, or has the effect of, increasing the recognition, appeal, and/or consumption of particular products and services” (11). Existing marketing platforms, such as television, radio, and outdoor advertisement channels (e.g., billboards, merchandise, and posters), are used to communicate messages and promote products and services to consumers (12). Promotional messages featured on these platforms are often packaged using persuasive marketing techniques deployed toward specific target individuals with common needs or characteristics (13). Such marketing techniques include the use of celebrity or sports endorsements, promotional characters, product claims, gifts/incentives, competitions, and games which are likely to appeal to children (14, 15). For children and adolescents, they are mostly exposed to such marketing techniques staged in media/settings (e.g., on television, schools, digital spaces, magazines, etc.) where they frequently utilize (16). They are particularly vulnerable because of their susceptibility to advertising techniques; they lack the cognitive ability to recognize the persuasive intent of advertising (17).

Currently, the literature on food marketing highlights the extensive advertising of less healthy options such as energy-dense foods and beverages, particularly sugar-sweetened beverages (12). Television food advertising remains the most researched advertising platform, although research on other media and settings is increasingly gaining attention. Reports from studies on outdoor food advertisements conducted in New Zealand (18), Australia (19, 20), and the USA (20) consistently show that most outdoor food advertisements are for unhealthy food or beverages, and they vary according to neighborhood characteristics. While data on outdoor food advertising in low-and middle-income countries is limited, accumulating evidence suggests a significant predominance of unhealthy food marketing in these countries. For instance, Chacon et al. found most the advertised food products around public schools in Guatemala were for sweetened beverages and soft drinks (21). In Mongolia and the Philippines, Kelly et al. reported over 85% prevalence of unhealthy food and drinks in the vicinity of schools (22). Furthermore, a recent study of outdoor food advertising around schools in Africa-Uganda recorded that over 80% of the food advertisements featured unhealthy food products (23). Our assessment of outdoor food advertising in the urban cities of Ghana also identified sugar-sweetened beverages as the most widespread food or beverage sold or advertised (24, 25).

Recognizing this evidence, the 63rd World Health Assembly endorsed a set of recommendations on the marketing of food and beverages to children (11). Member states were encouraged to use these recommendations to develop new and/or strengthen existing policies on food and non-alcoholic beverage marketing to children. However, in 2020, the WHO Global NCD Progress Monitor report indicated that Morocco is the only country within the African region to have fully implemented the WHO recommendation on the marketing of foods and non-alcoholic beverages to children (26). Currently, countries such as Chile and Spain are safeguarding children from unhealthy food environments through the promulgation of zoning laws prohibiting the promotion of unhealthy foods to children (27). In Ghana, food advertising is regulated by the Food and Drug Authority of the Ministry of Health. However, anecdotal evidence suggests that there is indiscriminate advertising of unhealthy foods and beverages within Ghanaian children's settings, including the immediate school environment. Empirical data is required to substantiate this assertion and guide public health initiatives and/or strategies aiming to improve children's food environment, including through policy development and implementation. This descriptive study aimed to examine the extent and nature of outdoor foods, and beverages advertising around selected schools in the Greater Accra region of Ghana.

## Materials and methods

### Study design

This descriptive cross-sectional survey is part of the MEALS4NCDs Project (28) which aims to measure and support public sector actions that create healthy food marketing, retail, and provisioning environments for children and adolescents in Ghana. The project adopted standardized frameworks, indicators, and tools to assess food promotion, food provision, and the Ghanaian community's readiness to support policy actions toward healthier food environments. The design of this current study draws substantially from the outdoor advertising protocol developed by the International Network for Food and Obesity/NCDs Research Monitoring and Action Support (INFORMAS) (29).

### Study location

**Figure 1:**
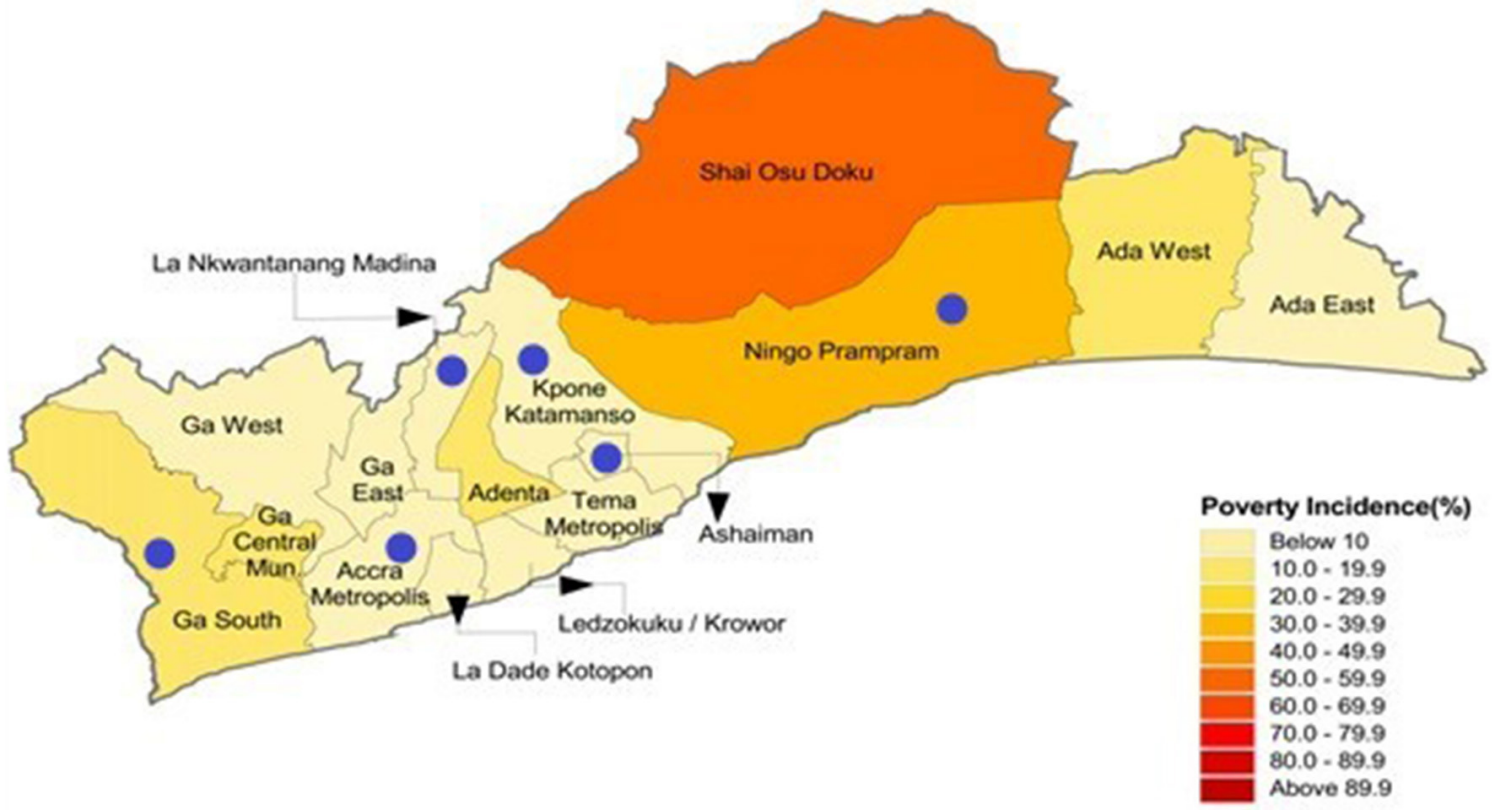
Map of the Greater Accra region of Ghana showing the various districts and poverty incidence.

Figure 1 displays the geographical location of the 16 administrative districts in the Greater Accra Region. The Greater Accra region was purposively selected out of Ghana's ten geographical regions (at the time the study was being conducted). It is the most populous and urbanized region in Ghana and hosts the capital city, Accra. The region has a cosmopolitan mix of cultures from all the other regions of Ghana. In 2018, the region had a total of 862 public primary schools and 812 public junior high schools, with a total enrolment of 431,782 (30).

A representative sample of six study districts was selected from the Greater Accra Region using a multistage sampling approach. We first grouped the 16 administrative districts within the Greater Accra region using the decentralized system of administration under the local government system of Ghana into three strata: Metropolitan, Municipal, and District. A metropolitan covers urban areas with populations of over 250,000. A municipal has a population of 95,000 or more, while a district covers a wider geographical area, combining rural areas and small towns, with a minimum population of 75,000 people. These administrative divisions are by themselves indications of the population density and urbanization status and give an idea of district-level “poverty incidence” (PI). The Ghana Statistical Service calculates and defines poverty incidence as the proportion of the population living below the national poverty line (6.6%). From each stratum, a random selection of Accra Metropolitan (PI: 2.6%) out of two metropolitans; Ningo Prampram District (PI: 31.2%) and Kpone Katamanso District (PI: 3.5%) from the five district assemblies; and La Nkwantanang Madina Municipal (PI: 2.8%), Ashaiman Municipal (PI: 4.4%), and Ga South Municipal (P1: 15.2%) from nine municipalities was performed.

### Schools sampling

Schools were the primary sampling unit (PSU) in this study. A sample of 200 schools representing about 25% of the schools within the region was selected from the six study districts. For each district, schools were stratified by levels (primary only, JHS only, and those having both JHS and primary levels). The required number of schools for each stratum from each district was calculated and sampled using proportionate sampling on the basis of the number of available schools for each level. This information was obtained from the Education Management Information System (EMIS) of the Ghana Education Service. Subsequently, a systematic random sampling technique was used to select the participating schools from each stratum after sorting using roll size data, defined as the number of pupils in the school. Full details on the sampling methodology have been published elsewhere (28).

### Data collection

For each selected school, zones earmarked for assessment were road networks within a distance of 250 m from the main entrance of each school. The 250 m distance is in accordance with the INFORMAS protocol (29) and has been used by previous studies investigating outdoor food advertising (19). The 250 m is considered a walkable distance that students can travel to make purchases during breaktime. Identification of the main entrance of each school was manually done by field supervisors with assistance from the head teachers after providing informed consent. All selected schools consented to participate in the study.

Data collection was carried out between July and August 2020 during weekdays (Monday–Friday, 9:00 a.m. to 5:00 p.m.) while schools were in session. This period ensured survey activities were conducted while full academic session was ongoing, allowing other components of the MEALS4NCDs project that required engagement with school authorities to be carried out. Prior to data collection, field research assistants with a minimum of a bachelor's degree were trained on using the data collection tool and study protocol. Emphasis was placed on the definition of an advertisement and what qualifies as a food (including alcoholic and non-alcoholic beverages) or non-food advertisement. Inter-coder reliability examination among field personnel was performed to check coding reliability after a pretesting exercise as part of the training. A total of 55 advertisements identified at a test site were independently coded by 12 research assistants. Data collected by the first author (GSA) was used as a reference, and percentage agreement was calculated for all field research assistants individually. Inter-code reliability calculated ranged from 85 to 90%. In this study, advertisements were defined in accordance with the INFORMAS Outdoor Advertising Protocol (29).

Six field teams (two-person teams) were each assigned to one of the six districts and tasked with conducting on-site visits and directly auditing all road networks in the dedicated zones at the selected schools for advertisements of food or non-food products. Assessment for each school zone was completed at a visit. Field workers used an observational checklist, designed using the Open Data Kit (ODK) application on an Android-based mobile phone with a built-in camera and geo-positioning functions, to objectively record descriptive information about each food advertisement. This format allowed simultaneous coding of advertisement characteristics (size of advertisement, setting of advertisement, type of advertisement, number of product types in the advertisement, product's name and brand, and the use of promotional characters and premium offers), photograph, and recording of the geo-location coordinates of the advertisements found. Effort was made to record the names of all products depicted in the advertisement in the event the advertisement promoted multiple products.

### Food classification

Classification of food products in advertisements was performed after the completion of field data collection. A separate training session was organized for three personnel (including GSA and AL) on how to correctly classify advertised food products. Inter-coder reliability testing was performed before commencing with the actual classification of the food advertisement. Each coder got a minimum of 80% inter-coder reliability on a test dataset.

Two different food classification systems, the NOVA classification system and the INFORMAS food classification system, were used to classify the foods and beverages advertised. With limited application in the Ghanaian setting, the two classification systems were adopted to ascertain how each evaluates the local food and beverage advertisements. The INFORMAS food classification system classifies food into “core/healthy”, “non-core/unhealthy” and “miscellaneous” foods. This classification system is based on defined cut-off points of fat and sugar (per 100 g of food). The cut-off points are different for each food group, taking into account differences in nutrient density. This food-based system encompasses 11 sub-food groups under core/healthy foods, 15 sub-food groups under non-core/unhealthy and 11 subgroups under miscellaneous. Core/healthy foods include fruits, vegetables, and water, while foods such as sweetened beverages, ice cream, and sweet biscuits are categorized as non-core/unhealthy foods. Miscellaneous foods include soup cubes, seasonings, and tea. Those advertisements with only a product/brand name or logo were classified separately. In this study, an advertisement was considered non-core/unhealthy if at least one food item in the advertisement was coded as non-core/unhealthy. This classification system has been used by previous studies, including some in Sub-Saharan Africa on outdoor food advertising (23, 24). The NOVA food classification, which is based on the nature, extent, and purposes of the industrial processes' foods are subjected, classifies foods into four groups: Unprocessed and minimally processed foods; processed culinary ingredients; processed foods; and ultra-processed foods and drinks products (31). Unprocessed and minimally processed foods include fresh and frozen vegetables, fruits, cereals, meats, poultry, and fish. Processed culinary ingredients include plant oils, animal fats, sugar and salt. Examples of processed foods include canned vegetables in brine, fruits in syrup, fish preserved in oil, while ultra-processed foods include ice cream, chocolates, candies, cookies, noodles, and carbonated drinks. Alcoholic drinks are not considered in this classification.

### Statistical analysis

Statistical software, IBM SPSS Statistics for Windows version 21, was used for data cleaning and statistical analysis. All recorded outdoor food advertisement data was used in the analysis. Descriptive analyses (frequencies, median, and Interquartile range) were used to summarize the number and characteristics of food advertisements, including those for promotional techniques (promotional characters and premium offers).

### Ethics

Permission to conduct this study was obtained in 2019 from the Greater Accra regional office as well as the participating district offices of the Ghana Education Service. Ethics approval was granted by the Ghana Health Service Ethics Review Committee (Approval # GHS-ERC 005-06-19) and the University of Ghana Ethics Committee for Humanities (Approval # ECH 152-18-19).

## Results

Assessment was conducted at all 200 sampled schools. Most of the schools were from the Accra metropolitan area (*n* = 54) with Ashaiman municipality recording the lowest number of sampled schools (*n* = 13). The “poverty incidence” across the selected districts ranged from 2.6% (lowest) for Accra Metropolitan to 31.2% (highest) for Ningo Prampram District.

### Extent of food advertisement in the areas around schools

**Figure 2:**
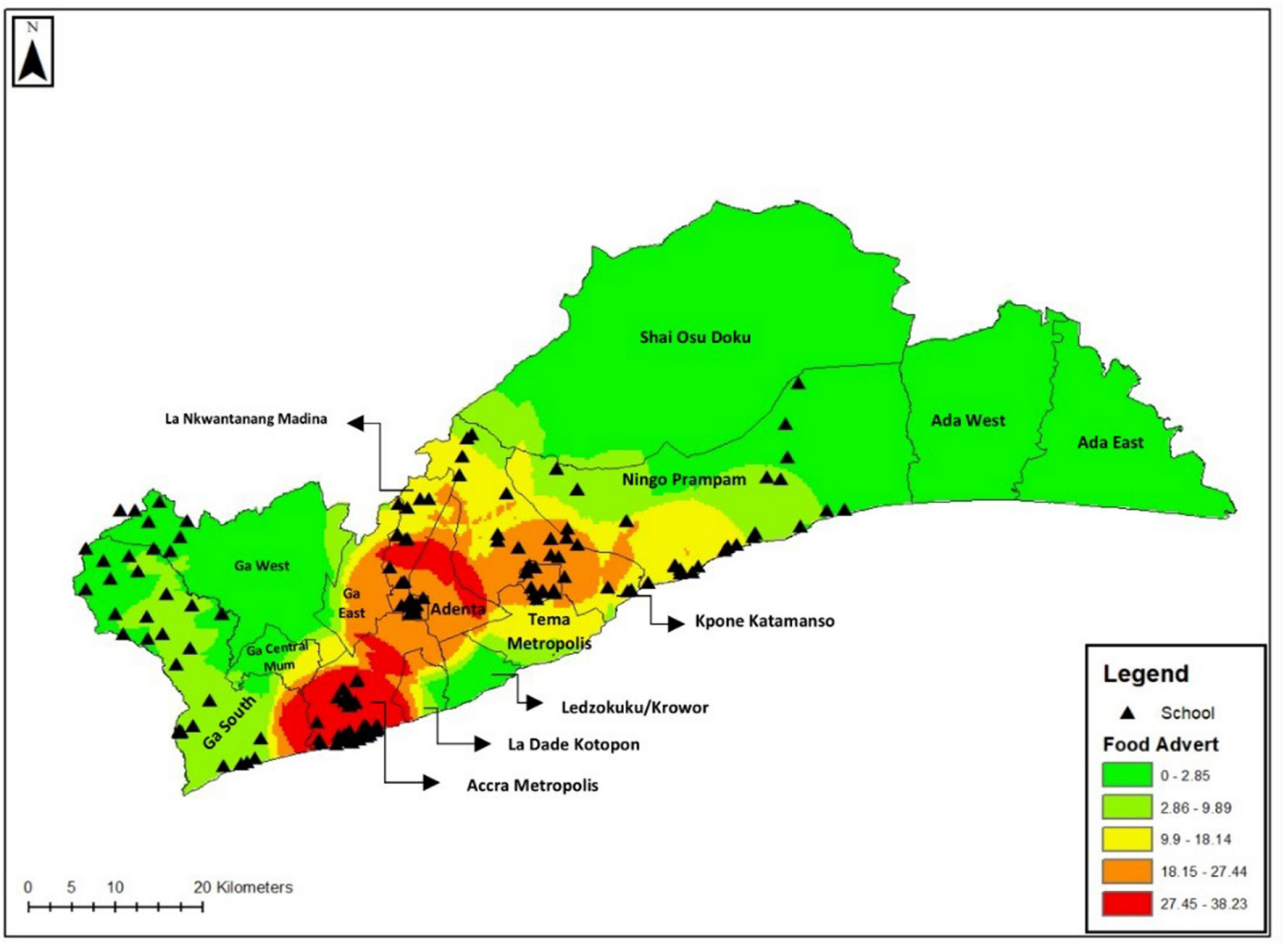
Hotspot map of advertisements recorded by district.

In total, 5,887 advertisements were identified, of which 2,469 (42%) were food-related. The number of food advertisements per school varied widely (range = 1–125) with a median number of 14 food advertisements. As shown in Figure 2, the proportion of food advertisements recorded in areas (districts) with a low poverty incidence was higher compared to those with a high poverty incidence. Food advertisements were more prevalent in the vicinity around of “JHS only” schools (median = 22) than in the vicinity of “Primary only” schools and Basic (having both primary and JHS units) schools (median= 11)—see Table 1.

**Table 1:** Median number of food and beverage advertisements along a 250 m road network around schools by school type and location characteristics.

	**Median food adverts**	**(25-, 75-percentiles)**
**School type**		
Primary school only (6–11 y)	11	(7, 22)
Junior high school only (12–15 y)	22	(12, 41)
Basic school (6–15 y)	11	(4, 22)
**School location**		
Accra metropolitan	19	(12, 41)
Ashaiman municipal	13	(8, 35)
Ga South municipal	4	(2, 7)
La Nkwantanang Madina municipal	23	(11, 47)
Kpone Katamanso district	16	(9, 33)
Ningo Prampram district	12	(5, 24)
**Location poverty incidence**		
High poverty incidence schools	6	(3, 14)
Low poverty incidence schools	20	(10, 39)

### Characteristics of foods and beverages advertisements

Overall, the majority (78%) of food advertisements were either within the premises of food shops (mostly convenience/provision shops) or attached to them. About 15% of adverts were by the roadside, and the remaining were posted on non-food buildings (6%) or in other settings (2%)—mobile stalls and bus shelters. Most food advertisements (71%) promoted a single food product, although 19% had two or more different products. About 10% of food advertisements display only the product/brand name or logo. Most food advertisements were in the form of posters or banners (71%). About half (49%) of the food advertisements were small in size, while over one-third (34%) were of medium size and 10% were large (Table 2).

**Table 2:** Characteristics of food advertisements in/around schools in Greater Accra.

	**Frequency (*n*)**	**Percent (%)**
**Setting of advertisement**		
Food shop	1,924	78
Road	364	15
Non-food building	140	6
Mobile stall	38	2
Bus Shelter	3	0
**Format of advertisement**		
Poster/banner	1,744	71
Merchandise (e.g., branded umbrella, branded fridge)	308	13
Free-standing sign	243	10
Painted building/wall	152	6
Billboard	21	1
**Number of product type in advertisement**		
Only product/brand name	244	10
Single food product type	1,746	71
Two or more food product types	479	19
**Size of advertisement**		
>A4 paper	177	7
Small (>A4 but <1.3 × 1.9 m)	1,203	449
Medium (>1.3 × 1.9 m but <2.0 × 2.5 m)	845	34
Large (>2 × 2.5 m)	244	10

### Types of advertised foods and beverages

**Figure 3:**
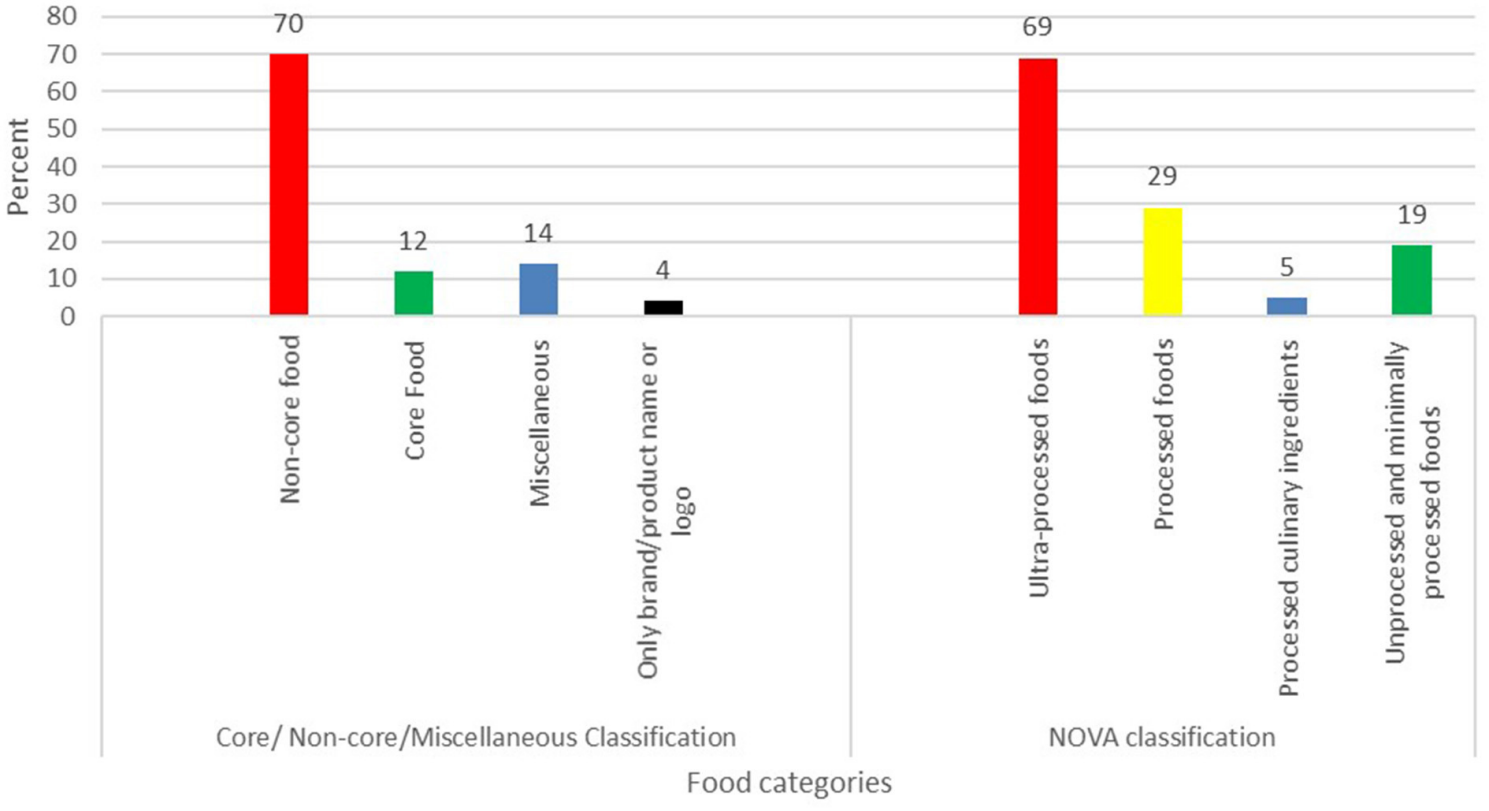
The proportion (%) of promoted foods and beverages by food categories.

Two-thirds (70%) of the food advertisements featured non-core/unhealthy foods. Core/healthy foods and miscellaneous foods contributed to 12 and 14% of food advertisements, respectively. About 4% of food and beverage advertisements had only a product/brand name or logo. The most frequently advertised food product subcategory was sugar-sweetened beverages (32%), followed by alcoholic beverages (12%) and high-fat and/or sugar-flavored dairy products and their alternatives (11%)—see Table 3. Four out of the top five most frequently advertised product subcategories belonged to the non-core/unhealthy food category. Bottled water (8%) was the most advertised product within the core food category. Advertisements for fruits and fruit products, and vegetables and vegetable products were rarely found (1 and 2%, respectively). About 69% of the foods and beverages advertised were ultra-processed foods; 29% were processed; 5% were processed culinary ingredients; and 19% were unprocessed or minimally processed foods according to the NOVA classification system (see Figure 3). The number of unhealthy food ads was higher around schools in the high poverty incidence area compared to the low poverty incidence urban area, with a median of 12 and 3, respectively (Table 4).

**Table 3:** Food categories and distribution by proportion of food advertisements in/around schools in Greater Accra.

	***N***	**%**
**FOOD CATEGORY**		
**Core foods**		
Bottled water	189	8
Meat and meat alternatives—include meat, poultry, fish, legumes and eggs	177	7.2
Rice/rice products without added fat, sugar or salt	143	5.8
Vegetables/vegetable products without added fats, sugars or salt	47	1.9
Low fat milks/yogurts and their alternatives	32	1.3
Fruits/fruit products without added sugar	31	1.3
Baby foods (exclude milk formulae)	28	1.1
Low fat/salt meals—include frozen or packaged meals	24	1.0
Low sugar and high fiber breakfast cereals	17	0.7
Oils high in mono- or polyunsaturated fats	15	0.6
**Non-core foods**		
Sugar sweetened beverages	799	32.4
Alcohol	302	12.2
High fat and/or sugar flavored dairy products and their alternatives	278	11.3
Meat and meat alternatives processed or preserved in salt	206	8.3
Ice cream, iced confection and desserts	130	5.3
Flavored/fried instant rice and noodle products	127	5.1
Sweet breads, biscuits, cakes, muffins, and high fat savory biscuits, pies and pastries	90	3.6
Fruit juice/drinks (<98% fruit)	64	2.6
Other high fat/salt products	57	2.3
Fast food (not only healthier options advertised), e.g., burgers, fries, soft drinks	56	2.3
Sweet/Savory snack foods	37	1.5
High sugar and/or low fiber breakfast cereals	17	0.7
Chocolate and candy	11	0.4
**Miscellaneous food**		
Local restaurant mixed dishes	446	18.1
Recipe additions (including soup cubes, oils, dried herbs and seasonings)	214	8.7
Tea and coffee (excluding sweetened powder-based teas or coffees)	31	1.3

Some advertisements depicted more than one food or beverage product.

**Table 4:** Median (25-, 75-percentiles) number of food and beverage category advertised by school characteristics.

	**Food category**		
	**Core**	**Non-core foods**	**Miscellaneous**
**School type**			
Primary school only (6–11 y)	4 (2, 4)	10 (5, 30)	3 (2, 6)
Junior high school only (12–15 y)	3 (1, 5)	12 (7, 35)	4 (2, 6)
Basic school (6–15 y)	2 (1, 4)	9 (3, 19)	2 (1, 4)
**School location**			
Accra metropolitan	3 (1, 4)	12 (9, 23)	3 (2, 6)
Ashaiman municipal	4 (2, 9)	4 (2, 9)	2 (1, 10)
Ga south municipal	2 (1, 3)	2 (1, 5)	1 (1, 3)
La Nkwantanang Madina municipal	4 (2, 6)	20 (9, 33)	4 (1, 7)
Kpone Katamanso district	4 (1, 5)	10 (5, 22)	2 (1, 5)
Ningo Prampram district	2 (1, 3)	9 (4, 19)	2 (1, 4)
**Location poverty incidence**			
High poverty incidence schools	4 (1, 5)	12 (8, 28)	4 (1, 6)
Low poverty incidence schools	2 (1, 3)	3 (2, 9)	1 (1, 4)

### Description of promotional techniques

**Figure 4:**
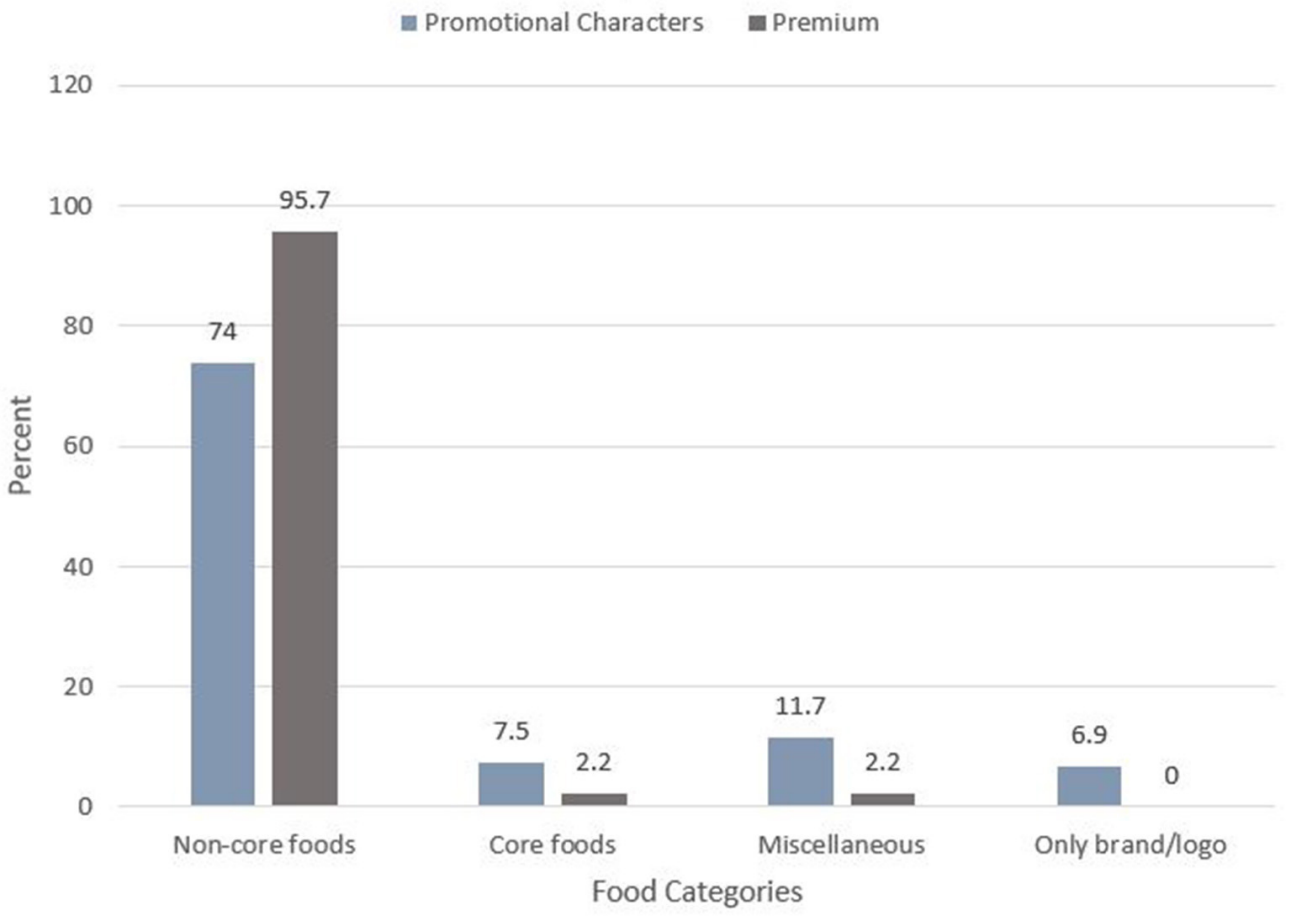
Promotional technique used on food advertisements by food categories.

Approximately 14% (*n* = 334) of food advertisements featured a promotional character. Cartoons/company owned characters (69%) and “for kids” images (23.4%) were the most predominantly used promotional characters compared to others, such as famous sports people, non-sport celebrities, and amateur sports people. Premium offers were present in 8% (*n* = 184) of all food advertisements. They include price discounts (28.8%), price promotions (60.9%) and “gifts and collectables” (7.6%). As shown in Figure 4, of the advertisements that featured a promotional technique, non-core foods advertised had the highest number of promotional characters and premium offers used (74 vs. 96%, respectively).

## Discussion

Tackling obesity and diet-related NCDs demands a multi-sectoral and multidisciplinary approach, including understanding environmental cues like advertising activities that promote the consumption of unhealthy foods. Findings from this study demonstrate that outdoor food advertisements (42%) are pervasive around schools in the Greater Accra region. Previous studies have reported varying densities of food advertisements at schools, citing neighborhood characteristics such as socioeconomic status and geographical (rural/urban) locations as contributing factors (32–34). Our study reveals s lower density of food advertisements in schools located in areas with high poverty incidence areas compared to those in low poverty incidence areas, which are more urbanized and have high population density neighborhoods. Food marketers gain more value (brand exposure) when food products are advertised in a high-density neighborhood due to the number of people that could be exposed to the advertisements as compared to the same activity in low-population areas.

Several studies have indicated the predominance of marketing of energy-dense, nutrient-poor foods and beverages around schools (18, 23, 24). The presence of unhealthy food advertising in and around schools can influence school children's food choices, considering the repeated exposure to these advertisements (35, 36). Such exposure can also contribute to what is referred to as the “normalization” of junk food (37, 38), as well as act as a cue for unhealthy food purchase and consumption (9).

The vast majority of food advertisements observed in our study promoted unhealthy food products, and most commonly sugar-sweetened beverages. This aligns with earlier research by Green et al. (25), which found that almost half of food and beverage advertisements in deprived urban neighborhoods in Ghana and Kenya were for sugar-sweetened beverages. In Uganda, a study that assessed food and beverage advertising surrounding schools in urban and peri-urban areas using the same methodology as the current study reported that 86% of food advertisements featured unhealthy products, of which sugar-sweetened beverages were again the most advertised product (23). The ubiquitous promotion of sugar-sweetened beverages around schools means school children are exposed to a large number of unhealthy food and beverage advertisements that can influence their intake. The dominance of sugar-sweetened beverage advertising as referenced in the aforementioned studies is consistent with reports of deliberate, relentless, and pronounced marketing activities of such products in this and other LMICs (39). Efforts aimed at improving population food environments and thus NCDs recommend the establishment of local or national policies that will limit the marketing of high-fat, salt, and sugar (HFSS) foods, especially to children (40, 41). It is important that these policies encompass tighter restrictions on sugar-sweetened beverages as they appear to be the most advertised food products.

Health researchers and practitioners have consistently raised concerns about the proliferation of unhealthy food marketing in settings frequented by children, including the school environment (42). This concern is precipitated by the increasing rates of overweight and obesity among children of school-going age (43). Considering that school nutrition interventions are implemented to positively promote healthy food choice and intake among school-going aged children, the presence of unhealthy food advertising may be a barrier to achieving the desired outcomes. In some countries, like South Korea, areas around schools are prohibited from food advertising practices to prevent school children exposure to unhealthy marketing practices (44). In the literature, it is evident that food-related activities within the environment where people live or spend most of their hours can influence their dietary patterns (45, 46). Basic schools in Ghana are typically situated within the community and are exposed to commercial activities, including the sale and advertisement of food products by food vendors operating in and around the school. Zoning initiatives that restrict unhealthy food promotion and availability and also promote the availability of healthy foods like fruits, and whole grain products could be useful to ensure a healthy school food environment in Ghana.

In our study, more than one in ten food advertisements surrounding schools were for alcoholic beverages. Elsewhere, exposure to outdoor alcohol advertising has been found to be positively associated with intentions to consume alcohol among school children (47). Further, higher exposure to outdoor alcoholic beverage advertising was found to be associated with higher intakes (48). The harmful use of alcohol is recognized by the WHO as a causal factor in more than 200 diseases, including NCDs and injury conditions (49). Like with other unhealthy foods and beverages, exposing children to marketing activities for alcohol could be detrimental to their wellbeing. In recent years, Ghana's Food and Drug Authority has developed guidelines for advertisements on foods and beverages, which include specific requirements for the advertisements of alcoholic beverages (50). The guidelines restrict the airing of alcohol advertising on radio and television between 08:00 a.m. and 08:00 p.m. It also prohibits the placement of alcohol advertising materials within 200 meters of schools. However, observations from this study show that outdoor advertisements, including posters and billboards, are used to promote alcohol within the school neighborhood, some of which were within the 200 meter prohibited zone. It is therefore important that future amendments to the guidelines should encompass restrictions for all outdoor advertising platforms in settings such as the school environment. At the time this study was undertaken, there were no specific national regulations to restrict the marketing of sugar-sweetened beverages to children, even though Ghana is a signatory to World Health Assembly Resolution 63.14, which sought to encourage efforts that would restrict the marketing of unhealthy food and non-alcoholic beverages to children (11). Clearly, results from this study show that efforts by regulatory bodies in Ghana need to be intensified.

Our study also found that posters and banners were the most used outdoor advertising type, accounting for over 70% of all advertisements recorded, although other channels like billboards, merchandise, and free-standing signs were also recorded. The usage of stationary materials like posters and banners as a means of advertising food has been reported to be heavily placed in close proximity to settings like schools and other places where they can be repeatedly seen by large numbers of people (4, 51, 52). For posters in particular, its attributes make them easily placeable onto any surfaces at multiple locations. Marketing researchers see this channel of advertisement as particularly impactful since it is embedded into the physical environment and people cannot avoid being exposed to it easily as compared to advertisements broadcast on platforms like television or radio (53). Therefore, efforts to regulate unhealthy food advertisements must be comprehensive in scope and should cover all advertising channels, including stationary outdoor advertisements.

The present study also showed that over three-quarters of food advertisements were placed at food outlets. This finding is suggestive that marketers exploit food outlets as marketing platforms. Available literature shows that promotional activities, especially in-store advertising at retail outlets, have the ability to influence consumer purchase by providing cues toward certain brands or products (54). Our assessment of in-store advertising is reported elsewhere (55). In their investigation of the retail food store exterior advertisements and the products sold in retail outlets, Barquera et al. found that about 60% of the advertised products were available at the food shops (4). For school children, food outlets within the school vicinity are places frequented during school hours, especially during break periods to purchase foods for consumption (56, 57). The presence of food advertisements at food outlets in the school environment can influence the purchasing behavior and consumption patterns as a result of repeated exposure (10). Children, in particular, are seen as vulnerable to marketing activities since they are not able to recognize the intent behind the advertisements (58, 59). Fernandes et al. reported that unhealthy foods are being sold to Ghanaian children in schools by private or independent vendors (59). Given that not all food outlets provide healthy food, the Ghana Education Service can put in place regulations to restrict the sale and promotion of foods having high sugar and fat content within the premises of the school compound. There is a need to extend this regulation out of the school premises to include the immediate surroundings accessible to school children since there is a chance for students to visit food outlets close to the school premises.

Regarding the link between promotion of unhealthy foods and health outcomes, most evidence relates to the effect of promotion on preferences and choices rather than on the ultimate adverse outcome, such as obesity. Some have, nevertheless, argued that, independent of other factors, exposure to unhealthy food marketing is a modifiable risk factor for obesity (60, 61). Kessler describes how sugar, fat, and salt activate neurons involved in taste perception, reward, and conscious control of eating. He theorizes that “chronic exposure to highly palatable foods changes our brains, conditioning us to seek continued stimulation. Over time, a powerful drive for sugar, fat, and salt competes with our conscious capacity to say no”. He identifies dopamine as one of the key neurotransmitters mediating the rewiring of brain circuits in this way.

## Contribution to knowledge

*Globally, poor diet is a major contributor to overweight and obesity among children and adolescents. Environmental factors influence availability of poor diets, and dietary habits. One such factors is food and beverage marketing. In the literature, food advertising, a form of food and beverage marketing, has been shown to primarily promote products high in fat, sugar, sodium, or salt content. Public health experts and international health organizations have advised governments and policymakers to restrict the marketing of unhealthy foods, particularly to children. However, research and monitoring reports show that unhealthy food marketing persists. Most of the data are from studies conducted in high-income countries. Such evidence is limited in the lower and middle income countries. Context-relevant data are required to inform regional and local guidelines, policies or regulations. The current study investigated the food and beverage advertising landscape in a lower middle-income country, Ghana, focusing on the school environment. The pervasive advertising of sugar-sweetened and alcoholic beverages as recorded in this study demands policy action to limit the exposure to children of unhealthy food advertisements*.

## Limitations

Having been purposively delimited to the Greater Accra Region, the results from this study may not be generalizable to other regions of Ghana. Any attempt to extrapolate the findings should recognize this limitation. Cross-sectional in design, this study could not detect seasonal variations in marketing practices.

## Conclusion

Overall, the data shows that school children in public sector basic schools in the Greater Accra Region of Ghana are exposed to unhealthy food advertisements, particularly sugar-sweetened beverages. There is a clear need for a national policy that restricts the advertisement of these protects, especially in children's settings.

## Data availability statement

The raw data supporting the conclusions of this article will be made available by the authors, without undue reservation.

## Ethics statement

The studies involving human participants were reviewed and approved by the Ghana Health Service Ethics review committee (Approval # GHS-ERC 005-06-19) and the University of Ghana Ethics Committee for Humanities (Approval # ECH 152-18-19).

## Author contributions

All authors listed, have made substantial, direct and intellectual contribution to the work. AL, MH, RA, CA, FZ, MEL, KM, DL, GA, and SV secured funding and contributed to research design. GSA, APA, WQ, SKA, and SN collected and analyzed the data. GSA drafted the manuscript. All authors reviewed and approved the final manuscript.

## Funding

This study was supported by funding from the International Development Research Centre (IDRC) Food, Environment, and Health Program—IDRC, Canada - Grant Number: 108983-001 (PI—AL). The funder, however, played no role in the study design, data collection, analysis or interpretation, or in writing the manuscript.

## Conflict of interest

The authors declare that the research was conducted in the absence of any commercial or financial relationships that could be construed as a potential conflict of interest.

## Publisher's note

All claims expressed in this article are solely those of the authors and do not necessarily represent those of their affiliated organizations, or those of the publisher, the editors and the reviewers. Any product that may be evaluated in this article, or claim that may be made by its manufacturer, is not guaranteed or endorsed by the publisher.
